# An Overview of Trichobacteriosis (Trichomycosis): An Underdiagnosed Disease

**DOI:** 10.7759/cureus.45964

**Published:** 2023-09-25

**Authors:** María Luisa Montes de Oca-Loyola, Paola Lumbán Ramírez, Fernando Gómez-Daza, Alexandro Bonifaz

**Affiliations:** 1 Dermatology, Hospital General de México Dr. Eduardo Liceaga, Ciudad de México, MEX; 2 Mycology, Laboratorio de Micología y Enfermedades Tropicales, Valencia, VEN

**Keywords:** axillary, corynebacterium flavescens, corynebacterium spp, trichobacteriosis, trichomycosis

## Abstract

Trichobacteriosis (trichomycosis) is an asymptomatic infection at the axillary hair level caused by a bacterium of *Corynebacterium spp.* The aim of this study is to identify the clinical, epidemiological, and microbiological characteristics of previously reported cases. A review was conducted including the cases of trichomycosis (trichobacteriosis) reported in the PubMed database up to June 2023. Twenty-nine articles were included, involving 365 patients in total. A higher incidence was observed in men, representing 94% of the cases, most of which were in the age range of 20-50 years. The most frequently affected clinical topography was the axillar (90% of the cases). Most of the patients presented change in hair texture and bromhidrosis, some other frequent clinical manifestations were hyperhidrosis, hair color change, and cromhidrosis; 6% of the patients were asymptomatic. The etiological agent most frequently identified was *Corynebacterium spp., *the flavescens variety being the most prevalent. The most common form of treatment was shaving and topical clindamycin. In conclusion, trichobacteriosis is an infection that most frequently affects men at the axillary level; it manifests clinically with few symptoms and usually has a good response to treatment.

## Introduction and background

Trichomycosis, also known as trichomycosis palmelina, is a disease once thought to be caused by a fungus [1]. It is caused by a bacterium of the genus *Corynebacterium*, so the term trichomycosis is inappropriate and the term trichobacteriosis should be adopted as it has been previously described by some authors [2,3].

Trichobacteriosis is usually caused by *Corynebacterium flavescens*, previously called *Corynebacterium tenuis*, which is responsible for the flava variant. Less frequently, the red and nigra variety occurs. Recently other bacteria apart from *Corynebacterium spp*. have been involved [1,2]. The *Corynebacteriaceae* family is part of the natural microbiota of the skin [2]. It is a disease that is more frequent in tropical and humid areas [1]. Some of the risk factors are hyperhidrosis, poor hygiene, increased local temperature, humidity, and obesity [1,2]. It is thought to be due to alterations in the apocrine glands. To manifest itself, there must be a balance between four factors: the speed of production of apocrine secretion, its viscosity, the speed at which the sweat dries, and the speed of hair growth to produce concretions [4,5]. In cultures where women are used to shaving underarm hair, it is more common in men. It is prevalent in adolescents and young adults and the most frequently clinical location affected is the axillary [6]. Other locations usually affected are the pubis and scalp. As clinical findings, we can observe sheaths and concretions that adhere to the hair shaft, most frequently yellow, coming to represent up to 95%-98% of the cases, and exceptionally red (rubra) or black (nigra) [1]. The infection is superficial in hair, localizing in the soft keratin [2]. It is usually asymptomatic, but sometimes patients complain of colored hair, colored clothing, bromhidrosis, or colored sweat. The diagnosis is mainly clinical, but we can use other tools to confirm the diagnosis. One that is accessible and easy to interpret is Wood's light fluorescence [2,3]. Some of the differential diagnoses are chromhidrosis, stone, trichorexis nodosa, pediculosis, monilethrix, and hair covering secondary to the use of products such as deodorants and powders [1]. The recommended treatment is shaving of the area and topical antibiotic [3]. The most used are fusidic acid and clindamycin. It is an easy entity to treat, but with a high rate of relapses. Recurrences are due to lack of adequate treatment. It is common when they only shave, without using antibiotics, which does not remove the infection, since when the hair grows again, the hair concretions form again because the bacteria persist at the base. So as such there is no time for the infection to return, but there is simply no healing and when the hair grows, the concretions are observed again. *Corynebacteria* family is also responsible for keratolysis punctata (which affects plants) and erythrasma (which affects inguinal and axillary folds). When these two entities coexist with trichobacteriosis in an individual at the same time, it is called the “corynebacterial triad” [2].

## Review

**Figure 1:**
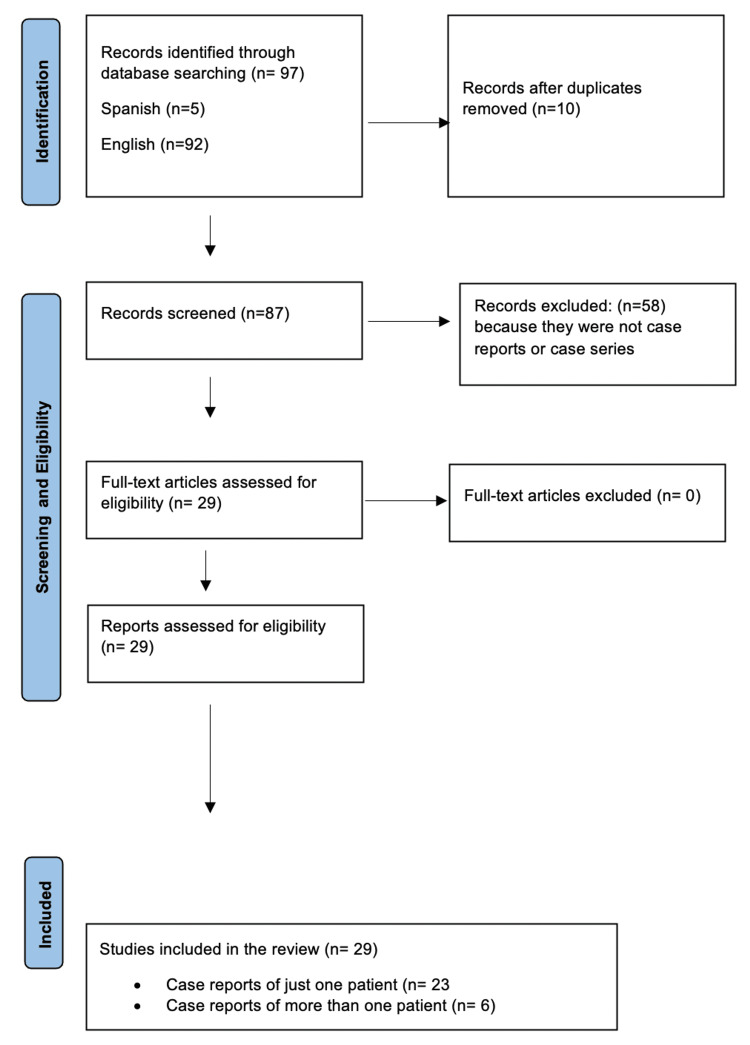
PRISMA flow diagram of the detailed process of selection of studies for inclusion in the systematic review

The literature review was systematically performed following the checklist of the Preferred Reporting Items for Systematic Reviews and Meta-analyses (PRISMA) guideline (Figure 1).

A search was conducted in the PubMed electronic database for articles published throughout the years up to June 2023 considering the terms “trichomycosis” and “trichobacteriosis,” as well as the Spanish terms “tricomicosis” and “tricobacteriosis.” A total of 97 records were obtained, although it was not possible to access some of them because they were too antique. The articles included were those reported in Spanish (n=5) and English (n=92). Duplicate studies were eliminated (n=10). Twenty-nine articles evidencing case reports were included and information was compiled such as author, date of publication, number of patients evaluated, gender, age, clinical presentation, clinical topography, symptoms, paraclinical tests (Wood’s light, KOH test, and dermoscopy), etiological agent and treatment.

Results

This review included 29 case report articles which comprised 365 patients. The demographic characteristics, associated symptoms, and microbiological findings of the study are shown in Tables 1, 2.

**Table 1:** Demographic characteristics and associated symptoms

Variables	N(%)
Sex	
Male	83/88 (94)
Female	5/88 (6)
Age	
<10 years	3/87 (3.5)
10-20 years	3/87 (3.5)
20-50 years	79/87 (91)
> 50 years	2/87 (2)
Clinical topography	
Axillar	321/ 358 (90)
Axillar + pubic	16/ 358 (4)
Pubic	4/ 358 (1)
Hair scalp	3/358 (0.8)
Axillar+ eyebrows	1/358 (0.3)
Symptoms	
Texture change	34/78 (44)
Bromhidrosis	30/78 (38)
Hyperhidrosis	5/78 (6)
Asymptomatic	5/78 (6)
Hair color change	5/78 (6)
Chromhidrosis	2/78 (3)
Pruritus	1/78 (1)

**Table 2:** Microbiological characteristics and treatments used *sl.: sensu latu

Variables	N (%)
Wood's light fluorescence in yellow concretions	
White	2/16 (12.5)
Yellow	8/16 (50)
Greenish yellow	2/16 (12.5)
Green	1/16 (6.25)
Greenish white	1/16 (6.25)
Yellowish white	1/16 (6.25)
Greyish white	1/16 (6.25)
Direct examination with KOH	
Concretions around the hair	66/66 (100)
Etiological agent	
* Corynebacterium flavescens*	56/71 (79)
Corynebacterium (sl.)*	12/71 (16.5)
*Corynebacterium rubra*	1/71 (1.5)
*Corynebacterium propinquum*	1/71 (1.5)
*Dermabacter hominis*	1/71(1.5)
Corynebacterium hominis	4/65 (6)
Treatment	
Shaved/Not shaved	
Shaved	10/19 (52)
Not shaved	9/19 (48)
Topic/Systemic	
Shaved + topic antibiotic	10/20 (50)
Shaved + clindamycin 1%	3/10 (30)
Shaved + erythromycin 2%	2/10 (20)
Shaved + benzoyl peroxide 5%	1/10 (10)
Shaved + fusidic acid 2%	2/10 (20)
Shaved + erythromycin 3% + clotrimazole powder	1/10 (10)
Shaved + aluminum chloride 15% + erythromycin	1/10 (10)
Topic antibiotic (alone)	8/20 (40)
Clindamycin	3/8 (37.5)
Fusidic acid	1/8 (12.5)
Clindamycin 1%+ ketoconazole shampoo	1/8 (12.5)
Clindamycin + benzoyl peroxide	1/8 (12.5)
Topical tetracycline	1/8 (12.5)
Naftifine 1%	1/8 (12.5)
Systemic antibiotic	2/20 (10)
Erythromycin + aluminum chloride + clotrimazole	2/2 (100)

**Figure 2:**
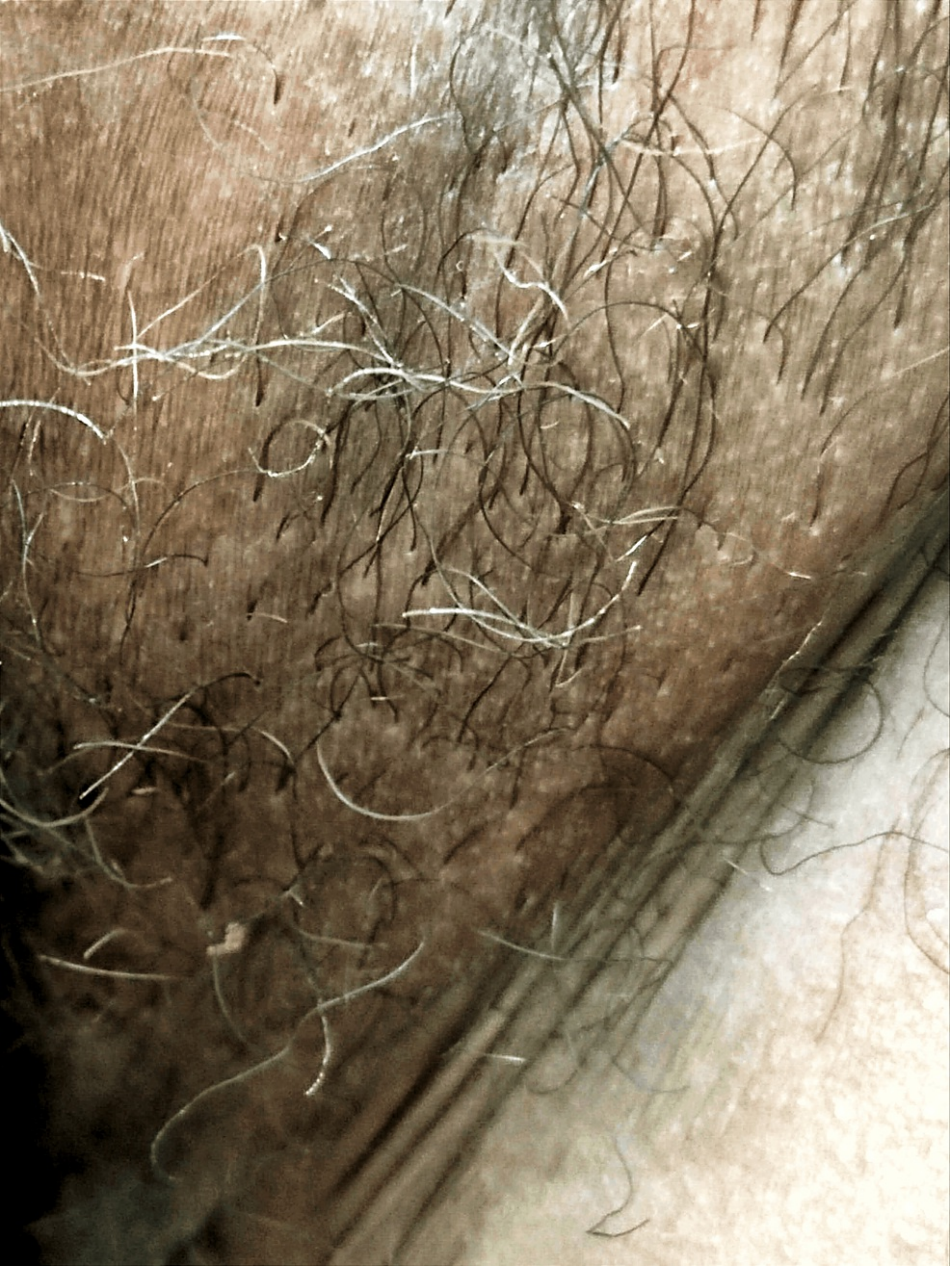
Trichobacteriosis, whitish hairs made up of bacterial concretions Image credit: Fernando Gómez-Daza

In 27 articles sex was reported, with a higher incidence in men 94% (83/88), with a 9:1 ratio. The average age was 20-50 years, representing 91% of the sample, 7% were younger than 20 years (6/87) and only 2% (2/87) were older than 50 years. The most frequently affected topography was the axilla (Figure 2), compromising 90% of cases (321/358), followed by axillar + mons pubis in 4% (16/358), mons pubis in 1% (4/358), the scalp in 0.8% (3/356) and axillar + eyebrows in 0.3% (1/358); in 5% (17/358) of the cases more than one area was involved.

**Figure 3:**
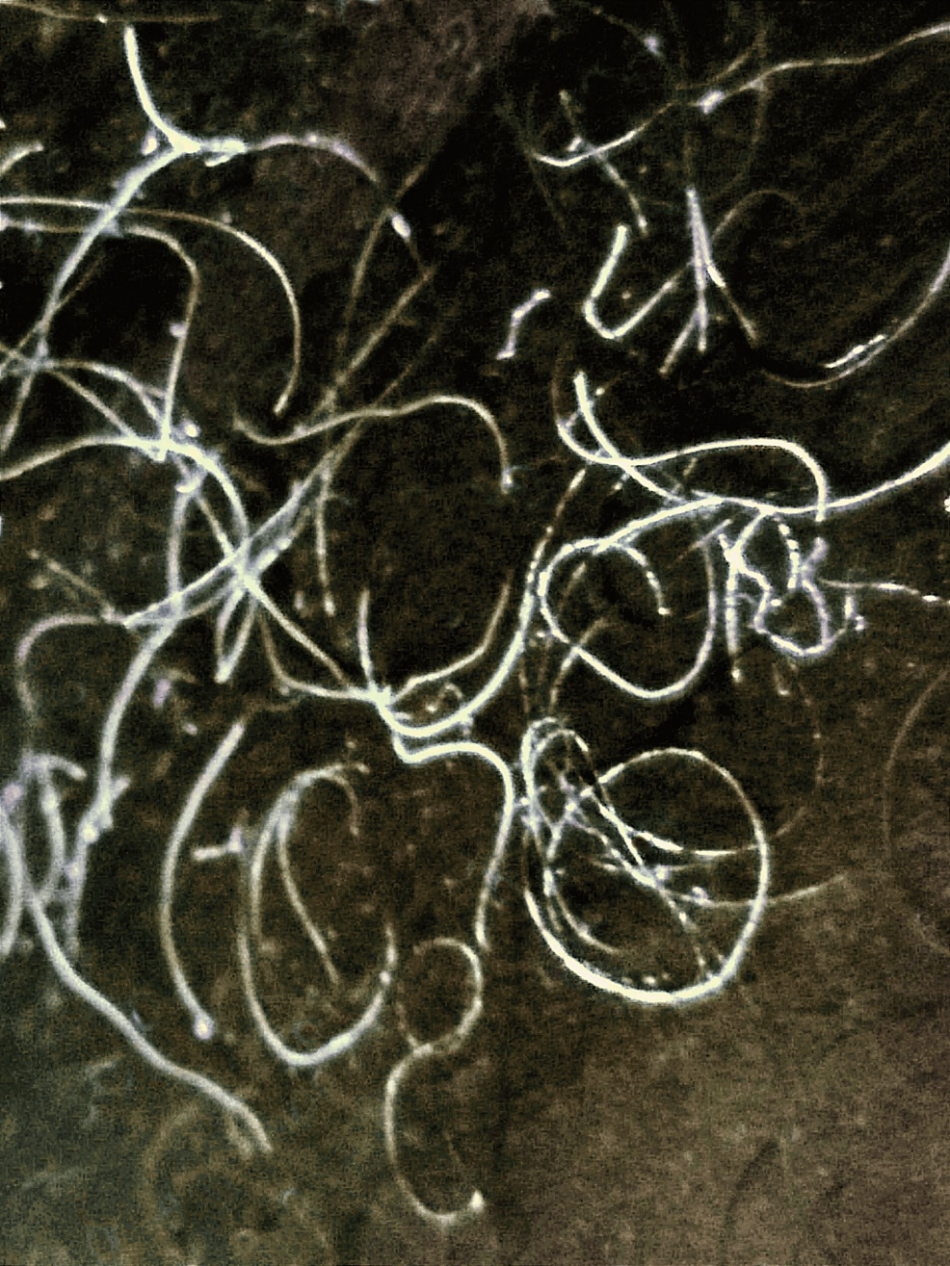
Trichobacteriosis concretions in Wood's light Image credit: Fernando Gómez-Daza

Other clinical presentations were hyperhidrosis in 6% (5/78), hair color change in 6% (5/78), cromhidrosis in 3% (2/78) and pruritus in 1% (1/78); 6% of the cases were asymptomatic (5/78) and in 78% (287/365) of the cases the symptoms were not specified. With Wood´s light, the most frequent fluorescence was yellow, representing 50% (8/16). Other fluorescence colors are white, greenish-yellow, green, greenish-white, yellowish-white, and greyish-white (Figure 3).

**Figure 4:**
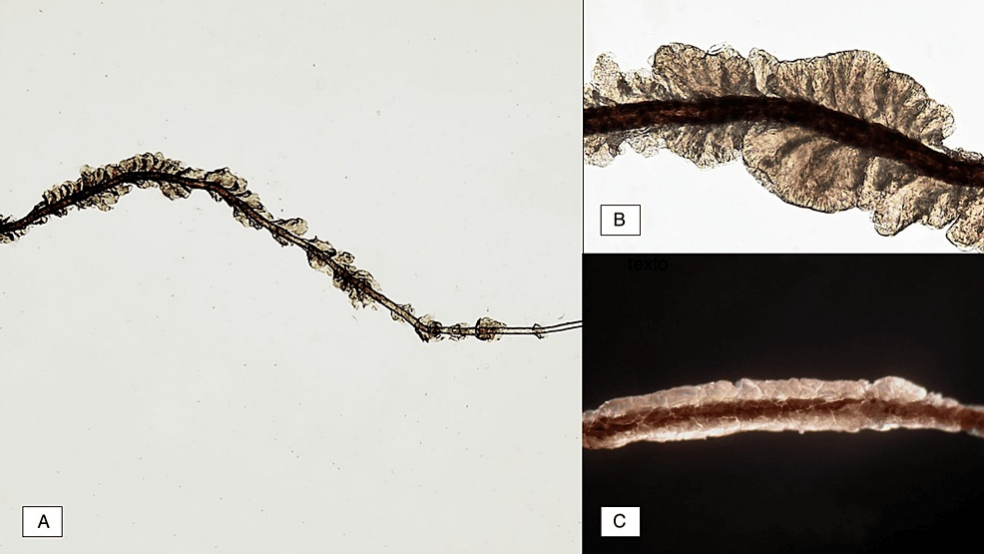
(A) Direct examination, overview of concretions of trichobacteriosis (KOH, 10x). (B) Close-up of concretions (KOH, 40x). (C) Trichobacteriosis with phase contrast microscopy (40x). Image credit: Fernando Gómez-Daza

In 100% of the patients, concretions were observed around the hair on direct examination with KOH (Figures 4A-4C). The most frequently identified etiologic agent was *Corynebacterium spp.*, of which the *C. flavescens* variety is the most prevalent. Only one case reported in the literature identified *Dermabacter hominis* as the causal agent. The most frequently used treatment was shaving the area and topical clindamycin 1% with excellent therapeutic outcomes.

Discussion

Trichobacteriosis was first described by Paxton in 1869, in an article entitled “On a disease condition of the hairs of the axilla, probably of parasitic origin” [7,8]. Named for its similarity to *Trichophyton tonsurans* [8]. Paxton thought that he had detected spores like those found in favus or tinea favosa but was unable to find the mycelial structures [7]. Later, in 1927, Castellani observed that the flava variety was apparently caused by “a very thin fungus,” with a bacillary appearance, which he named *Nocardia tenuis*, but he was unable to cultivate it [9,10]. He attributed the rubra and nigra varieties to a symbiosis of this fungus with chromogenic cocci; a black pigment-producing cocci (*Mycrococcus nigricans*) in the nigra variety and red pigment-producing cocci in the rubra variety [11,12]. In 1952, Crissey et al., after studying 100 consecutive cases in a University Hospital in Philadelphia, where 23 patients had clinical evidence of trichobacteriosis, identified that it was caused by a single diphtheroid, which they cultured and named *C. tenuis* [13,14]. They considered that there was nothing in their findings that would lead to classify this organism in the *Actinomycetale*s, even though there were many similarities between *Corynebacteria* and *Nocardia* species since they did not observe the ramifications previously described. Therefore, they considered that the cause of trichobacteriosis was due to a diphtheroid, rather than *Nocardia* [13]. Montes discovered that the morphology and structure of the organism resembled other diphtheroids, such as the erythrasma-producing *Corynebacterium minutissimum* [15]. Freeman et al., after studying seven patients, demonstrated that trichobacteriosis may be produced by at least three species of *Corynebacteria* [8]. In 1963 Shehadeh and Kligman suggested that the diphtheroids associated with trichobacteriosis are the usual microbiota of the axilla [12]. In 2014, *C. propinquum*, which had not been previously reported, was cultured in a patient, which is a bacillus of the normal microbiota of the oral mucosa in humans [14].

The infection is caused by a bacterium of the genus *Corynebacterium*, which is the most frequent variety of *flavescens*, as we could see in our study, which represents 80% (57/71), although many of the reports included did not identify the etiological agent, which could represent a higher proportion. The rubra variety is the one that follows in frequency, and lastly the nigra, with very few cases reported in the literature. This is a Gram-positive, aerobic, coryneform-actinomycete bacterium, composed of bacillary and diphtheroid forms [16,17]. The family of these bacteria is usually part of the skin microbiota, representing around 50% [2,18]. Other agents involved are *Corynebacterium propinquum* or *Serratia marcenscens* [6,9]. Recently a case reported by Larrondo et al. in 2017 caused by *D. hominis *was described, which is also part of the usual skin microbiota [19].

It is more frequent in tropical and humid areas [1]. The risk factors for transmission are increased local temperature, humidity, obesity, and poor hygiene [8,20]. Transmission from person to person has also been described in very close groups such as soldiers and athletes [6]. It is more prevalent in adolescents and young adults [6]. As shown in the table, the average age that is mostly affected is between 20 and 50 years of age, representing 91% of the cases. This is due to the higher apocrine secretion, which is considered key for the disease to occur. However, it is noteworthy that there are also cases reported in the pediatric age group, when there is still no activity of the apocrine glands, presenting on the scalp, where the number of apocrine glands is scarce or null. Therefore, we consider that the etiopathogenesis is not yet well clarified and that other mechanisms may be involved [17,21].

Infection begins when the etiological agent encounters the hair shaft and the bacteria adheres to the hair cuticle, using a cement-like substance, which is an insoluble substance [6]. The encapsulated corynebacteria creates a biofilm, which helps to evade the immune system of the host [3]. There is a strong association between the lipids on the hair surface and the physical properties of the hair initiating an attraction to the bacteria in conjunction with other factors to produce cuticle destruction [22]. Electron microscopy has demonstrated that the microorganism does not penetrate the medullary cortex of the hair, but it rather adheres very well to the hair surface and slowly develops until it forms concretions [6].

Levit suggests that the cement is synthesized by the apocrine glands of the host and by the microorganism [6], which explains the most affected topography, the axillary, 90% (321/358), representing 95% in previous reports, which shows an adequate correlation with our study. Mons pubis and the scalp in a smaller proportion [21]. On the other hand, Wilson et al. consider that it is more of a coincidence that trichobacteriosis affects the axilla more than other areas since it would otherwise affect the perineum more frequently [23]. Other authors consider that the axilla is the preferred topography since it is more exposed to bacteria, whereas factors such as deficient hygiene and humidity of the area are more relevant for the establishment of the disease [17].

It is an underdiagnosed disease since it presents with banal symptoms or may be asymptomatic, patients do not seek medical attention, so it may be more common than it appears [6,9]. In 2013, Bonifaz et al. reported a series of 56 cases with epidemiological, clinical, and microbiological findings [6]. They observed that up to 97.4% of patients had axillary involvement [6]. A case was reported where, in addition to having axillary involvement, there was involvement of the eyebrows, which had not been previously reported. It is believed that it was due to autoinoculation [6]. The flava variety was observed in 98% of the cases [6].

Trichobacteriosis is most prevalent in men [6], representing up to 94%, with a 9:1 ratio, which can be explained by the fact that women tend to shave this area, which diminishes the risk. Another reason why it prevails in men is due to physiological gender differences that influence skin properties which include hormone production, sweating rate, sebum production, surface pH, and skin thickness and hair growth, which are also factors that determine de number and composition of the skin microbiota. It has also been shown that adult males carry higher populations of bacteria on the skin, generally because the micro-colony size is larger in males than in females [24].

Levit mentions in this regard that it is true that trichobacteriosis does not appear in all armpits with the presence of the *Corynebacterium* microorganism and apocrine secretion [4]. He considers that there must be a balance in four factors: the speed of production of apocrine secretion, its viscosity, the speed at which the sweat dries, and the speed of hair growth to produce concretions [4,5].

Involvement of more than one area is rare since the bacteria are trapped in the cement-like substance [5]. They represent only 5% of cases (17/358). For this reason, it is also very difficult to transmit it from person to person, but it has been described in closed groups and overcrowded areas [5,17].

From the clinical point of view, we can observe mucoid sheaths and irregular yellow concretions (flava) in 95%-98%, and on rare occasions red (rubra) or black (nigra), which adhere to the hair shaft [8,17,23]. The root and adjacent skin are usually spared, but the damage extends along the entire hair; although in some cases it affects a smaller area [1,7]. The most common variety is yellow and the least frequent is black [8]. It is unknown whether the different colors are due to variations in the *corynebacteria* or to pigment-producing cocci [8]. The infection is superficial in the hair, targeting the soft keratin [6].

Initially, the concretions are invisible, only a thickening of the hair on palpation is perceived [6]. Then some masses are formed independently [6]. As the infection becomes chronic, the concretions spread along the hair until they form sheaths, causing thickening of the hair [6]. The associated manifestations are diverse, but they can also be asymptomatic, being observed in 6% of patients [8]. The most common manifestations are the change of texture and bromhidrosis, occurring in 44% and 38% respectively. Other associated symptoms are hyperhidrosis, color change, chromhidrosis, and pruritus, occurring in 6%, 6%, 3%, and 1% of our patients, respectively [8]. In general, the secretion of the apocrine glands is colorless and odorless, but it is modified by bacteria [23].

The diagnosis is mainly clinical, but there are other tools that are helpful in confirming the diagnosis, such as Wood's light, dermoscopy, Gram staining, and cultures [20]. Fluorescence with Wood's light is variable (yellow, red, or black), being yellow the most frequent [20]. In the case of being produced by *S. marcenscens* it does not fluoresce [9]. It is easy to make the clinical diagnosis when it affects the axillary area, but when it affects less frequent topographies such as the scalp or mons pubis, there are several differential diagnoses macroscopically, for this reason, we can rely on Wood´s light, and dermoscopy [21]. In trichobacteriosis, using polarized-dermoscopy we can observe concretions or sheaths around the hair, of waxy appearance [1,18,21]. Some authors have called them the feather and skewer signs [1]. In addition, concretions with the appearance of a “rosary of crystalline stones” are described [1]. Direct mycological examination shows an opaque material covering the hair fiber, without invasion of the cortex [25]. The gold standard is the observation of bacterial concretions in the hair and relating it to the culture [20]. Rich cultures (blood agar, BHI) should be used, where the microorganisms are observed as bifurcated coryneforms (like “drumsticks”) and their biochemical identification most often corresponds to *C. flavescens* [17]. In recent years, another species was identified by molecular biology, named *C. propinquum*, indicating that there may be other agents causing this infection [1]. API-Coryne® is a standardized system for the identification of coryneform bacteria in 24 hours, with an identification rate of 91% [9,14].

There are several differential diagnoses. The change of coloration may lead us to suspect chromhidrosis, which is an alteration of the apocrine glands [8]. Other differential diagnoses to consider are piedra, trichorrhexis nodosa, pediculosis, molinetrix, and hair coating secondary to the use of products such as deodorants and talcum powder [8,20].

Shaving of the affected area is recommended, but it is not sufficient [8]. It is treated with topical treatment based on benzoic acid, salicylic acid, and antibiotics such as fusidic acid, erythromycin, and clindamycin [6,8]. The most used treatment in the literature is shaving of the area and topical clindamycin at 1% as we could see in our table. In our experience, axillary cases respond better to shaving of the area in combination with fusidic acid [17]. Treatment is not complicated, but the condition tends to recur when it is only treated with shaving [8]. Recurrences are due to a lack of adequate treatment. It is common when they only shave, without using antibiotics, which does not remove the infection, since when the hair grows again, the concretions form again because the bacteria persist at the base. As such, there is no time for the infection to return, but there is simply no healing and when the hair grows, the concretions are observed again.

## Conclusions

The research allowed us to corroborate the data previously reported in the literature. We found a higher frequency of trichobacteriosis in young men, with predominantly axillary involvement. The less frequent cases reported in childhood tend to present on the scalp. The most reported clinical manifestation is textural change and bromhidrosis. The etiologic agent is under-reported in the literature, but we agree that *C. flavescens* is the most frequent. Finally, we were able to conclude that the most used treatment is shaving of the affected areas and topical clindamycin, with good clinical results.

Trichobacteriosis is an underdiagnosed disease that is more prevalent in patients living in humid areas. This is due to the few manifestations it gives and the shaving habit in women. Despite the few manifestations, we carry out a good clinical examination, since it is an easy disease to treat, with a good prognosis and a tendency to recur.
